# Identification of a Novel Retinoid by Small Molecule Screening with Zebrafish Embryos

**DOI:** 10.1371/journal.pone.0001947

**Published:** 2008-04-09

**Authors:** Chetana Sachidanandan, Jing-Ruey J. Yeh, Quinn P. Peterson, Randall T. Peterson

**Affiliations:** Cardiovascular Research Center, Massachusetts General Hospital, Harvard Medical School, Charlestown, Massachusetts, United States of America; University of Washington, United States of America

## Abstract

Small molecules have played an important role in delineating molecular pathways involved in embryonic development and disease pathology. The need for novel small molecule modulators of biological processes has driven a number of targeted screens on large diverse libraries. However, due to the specific focus of such screens, the majority of the bioactive potential of these libraries remains unharnessed. In order to identify a higher proportion of compounds with interesting biological activities, we screened a diverse synthetic library for compounds that perturb the development of any of the multiple organs in zebrafish embryos. We identified small molecules that affect the development of a variety of structures such as heart, vasculature, brain, and body-axis. We utilized the previously known role of retinoic acid in anterior-posterior (A–P) patterning to identify the target of DTAB, a compound that caused A–P axis shortening in the zebrafish embryo. We show that DTAB is a retinoid with selective activity towards retinoic acid receptors γ and β. Thus, conducting zebrafish developmental screens using small molecules will not only enable the identification of compounds with diverse biological activities in a large chemical library but may also facilitate the identification of the target pathways of these biologically active molecules.

## Introduction

Small molecules have proved to be powerful tools for understanding the importance of molecular pathways in development and disease. When compared to traditional genetic mutations, they have the advantage that they can be used to reversibly perturb the functions of molecules at different points of embryonic development or disease progression. Due to the concentration-dependent effects of small molecules on their targets, it is also possible to uncover phenotypes that result from partial inactivation of a pathway. Their intracellular permeability permits their use in cultured cells as well as whole organisms and makes them candidates for use in therapeutics. This has made the identification of novel small molecule modulators of important genetic networks an area of intense interest.

In an effort to discover novel disease modulators, a number of synthetic and natural chemical libraries have been assembled in recent years [1]–[3]. The relative simplicity of purified proteins, cultured cells, and other *in vitro* systems has been utilized successfully in many targeted small molecule screens, resulting in the identification of novel biological tools and therapeutic candidates [4], [5]. However, such screens interrogate a library for very specific interactions with one or a few protein targets, and hence only the small percentage of the library with one specific activity is uncovered. Thus, to explore the full potential of a diverse chemical library, a vast number of targeted screens would be essential. In contrast, the use of a complex but sensitive assay capable of identifying perturbations in thousands of potential targets simultaneously affords the possibility of uncovering a large proportion of all unknown modulators of biological pathways contained in these libraries. Zebrafish embryos make an ideal model system for in vivo small molecule screens due to their small size, optical transparency, accessibility during development *ex utero*, and permeability to small molecules [6]. Moreover, since embryonic development and patterning require the precise regulation of myriad different genetic networks, they are extremely sensitive to perturbations by small molecule modulators.

A major challenge of employing phenotypic screens for identifying small molecules is delineating their molecular targets. Zebrafish embryogenesis progresses through a strictly stereotypical sequence of events choreographed by numerous signaling pathways. Perturbation of these molecular pathways leads to distinct and predictable defects in the embryos that are easily detectable and have been extensively characterized for thousands of genetic mutants (www.zfin.org). Therefore, in many cases, the developmental defects caused by a small molecule can be linked with high resolution to a specific genetic pathway known to cause the same defect.

Many biological pathways that govern embryonic development remain active in the adult, and the study of embryonic development has led to important insights into the role of these pathways in adult physiology and perturbations that lead to disease. Therefore, small molecule modulators of gene networks active in early development can lead to a better understanding of, and design of therapeutics for, adult diseases.

Large-scale genetic screens in zebrafish have traditionally not focused on a specific biological process but rather have searched broadly for all mutations causing visible phenotypes of any kind. These screens have identified phenotypes ranging from subtle morphological defects of the ear to cardiac arrhythmias. Subsequently, positional cloning has linked many of these phenotypes to specific gene mutations. In a similar way, zebrafish small molecule screens can interrogate a small molecule library broadly for all compounds causing visible phenotypes of any kind, and comparison to known mutant phenotypes can link specific small molecules with genes that cause similar phenotypes. We screened a small molecule library for compounds that affect patterning and organ development in zebrafish embryos. We have identified more than 100 compounds that perturb a number of different aspects of development, including body-axis, brain, heart, and vascular development. In this report, we examine one of these compounds, DTAB, as an illustration of how zebrafish phenotypes can connect small molecules with their molecular targets. DTAB was identified for its ability to cause an anterior-posterior (A–P) axis-patterning defect in the embryos. Guided by the known role of retinoic acid (RA) in A–P patterning in embryos we were able to show that DTAB exhibits a retinoid-like activity and is able to phenocopy the effects of RA treatment on zebrafish embryos. Using receptor activity assays, we demonstrate that DTAB is also able to activate the retinoic acid receptors (RARs and RXRs) with selectivity for RAR-γ.

## Results

### Developmental screen for small molecule modulators of development

**Figure 1:**
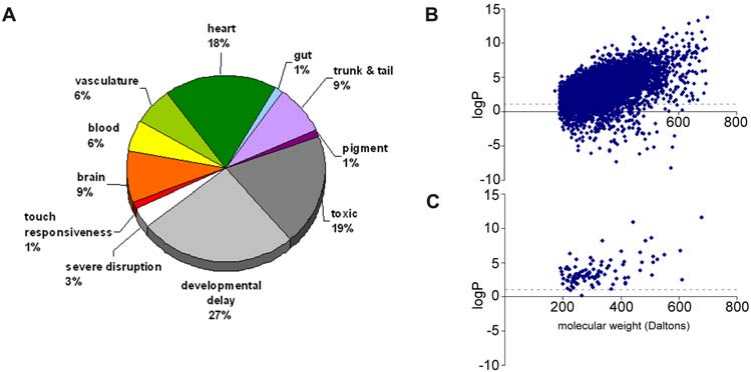
Zebrafish small-molecule screen identifies compounds that effect development. a) 4% of the 5760 compounds screened affected zebrafish embryos. 19% of the active compounds caused lethality. 175 compounds with detectable effects on zebrafish development were identified. b–c) The small molecules used for screening had molecular weights ranging from approx. 200 daltons to 700 daltons and had log P (partition coefficient) values ranging from −10 to +15. However, the log P of the bioactive compounds were restricted to 0 to +12. b) log P values between octanol and water of all the compounds screened plotted against their molecular weights. c) log P values of all the bioactive compounds (excluding the toxic compounds) plotted against their molecular weights.

**Figure 2:**
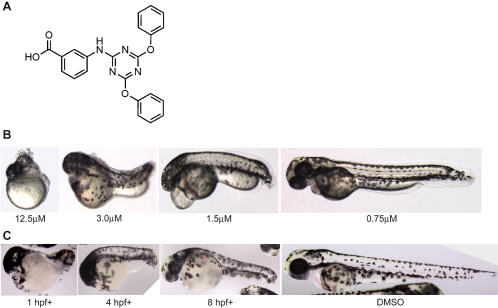
DTAB causes anterior-posterior axis defects. a) Chemical structure of DTAB. b) Embryos treated with higher concentrations of DTAB showed more anterior-posterior patterning defects than those treated with lower concentrations. Zebrafish embryos were treated from 1hpf to 48hpf with different concentrations of DTAB. c) Embryos treated earlier in development, and thus for longer duration were more severely affected than those treated later. Embryos were exposed to 3 µM DTAB from different times of development to 48hpf and compared to embryos treated with the vehicle DMSO. Vehicle (DMSO) treated controls developed normally. All observations were made at 48hpf.

To identify small molecule modulators of development, 5760 compounds from a diverse synthetic chemical library (Chembridge Corporation, Diverse set E) were screened. Embryos at 1–4 hours post fertilization (hpf) were treated with the compounds and scored for obvious developmental defects in the body-axis, brain, heart, blood and vasculature at 24, 48 and 72 hours. 5540 compounds out of the 5760 tested (96%) caused no discernible phenotype at 72hpf and 45 compounds (0.8%) resulted in toxicity leading to death. 175 (3%) compounds affected normal development of the zebrafish embryos. Of these, 61 caused general developmental delay or embryos failed to hatch out of the chorion and 8 compounds caused severe disruption of multiple structures during development. The remaining 106 compounds caused phenotypes that included effects on the brain (21), heart morphology and function (43), blood and vasculature (28), gut (3), trunk and tail (20), pigment formation (3), and touch responsiveness (3) (Fig. 1A). The small molecule DTAB (IUPAC: 3-[(4,6-diphenoxy-1,3,5-triazin-2-yl)amino]benzoic acid) (Fig. 2A), caused a shortening of the body axis.

### Biologically active compounds have positive log P values

Since 96% of the compounds in the library did not appear to have any effect on embryogenesis we compared the chemical properties of the active and inactive compounds to identify properties that might determine biological activity. The ability of a compound to produce a phenotype in embryos depends on its physico-chemical properties as well as its interaction with developmentally relevant molecules. In terms of their size distribution the active compounds exhibited no bias and included the full size-range of the library, from 200 daltons (Da) to 700 Da (Fig. 1B–C). The partition coefficient between octanol and water (logP) of the total library screened ranged from −10 to +15, thus containing both hydrophilic and hydrophobic compounds (Fig. 1B). However, the logP of the active compounds clustered between 0 and +15 with a marked absence of hydrophilic compounds. Within the hydrophobic range, there appeared to be no bias (Fig. 1C). Thus we speculate that hydrophilic compounds, as measured by a negative log P value are rarely active in embryonic zebrafish screens, perhaps because they penetrate zebrafish embryos less effectively.

### DTAB causes a shortening of anterior-posterior axis

Treatment of embryos with DTAB (12.5 µM) at 1hpf caused a severe patterning defect resulting in embryos with almost no identifiable morphological features except scattered melanocytes across the skin surface (Fig. 2B). At 3 µM the head and tail structures were still reduced but a number of somite boundaries were apparent. At 1.5 µM there was evidence of head structures with brain folds and poorly developed eyes. There was no blood circulation and embryos exhibited pericardial edema. At the lowest concentration tested (0.75 µM) the A–P axis was extended to nearly normal lengths and anatomical structures appeared normal. Blood circulation was also normal.

In order to determine if the molecular target of DTAB was functional early or late in development, the embryos were exposed to 3 µM DTAB at different developmental stages from 1hpf to 24hpf (Fig. 2C). DTAB appeared to affect development at all stages, but embryos exposed later in development exhibited weaker phenotypes, indicating a cumulative effect over time. Severity of the phenotype was also a function of the length of exposure. Embryos exposed for longer time periods showed more severe phenotypes compared to those incubated in the compound for shorter time intervals followed by removal of the compound from the water (data not shown).

**Figure 3:**
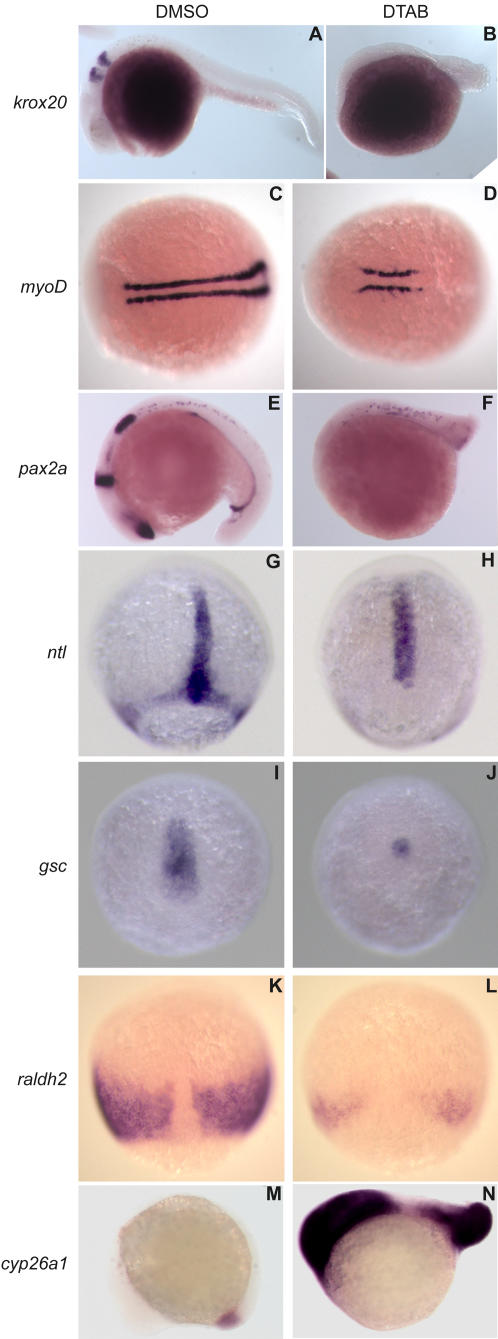
DTAB affects the expression of early developmental markers. *In situ* hybridizations of zebrafish embryos show that treatment with DTAB results in severe reduction of *krox20*, *pax2a*, *myoD*, *ntl*, *gsc*, and *raldh2* staining. DTAB treated embryos exhibited an upregulation of *cyp26a1* transcripts in the tail and head. Embryos were treated with 12.5 µM DTAB or DMSO control from 4hpf onwards until fixation. Embryos were fixed at 75% epiboly (G–L), 10-somite (M–N), 18-somite (C–F) and 26-somite (A,B) stages. A–B, E–F, M–N are lateral views with anterior to the left and dorsal to the top. C–D are dorsal views with anterior to the left. G–L are dorsal views with the animal pole to the top.

*In situ* hybridization for early developmental markers was performed in order to elucidate the molecular nature of the short-axis phenotype. Dorso-ventral patterning defects result in short anterior or posterior structures [7]. So, we analyzed the expression of *krox20*, a marker for dorsally derived hindbrain segments rhombomere 3 and 5. *Krox20* expression was completely absent in embryos treated with 12.5 µM of DTAB at 4hpf onwards (Fig. 3 A–B). *MyoD* expression indicated that myoblasts were correctly specified in the emerging somites, however there was a severe reduction in the total number of somites compared to untreated embryos further confirming the general shortening of the anterior-posterior (A–P) axis in the embryos (Fig. 3C–D). Staining for *pax2.1* in ventral structures such as pronephric ducts and dorsal structures like the midbrain-hindbrain boundary, optic stalk and otic vesicle were also absent, with only residual staining in the spinal chord neurons (Fig. 3E–F). Thus, DTAB was able to ablate both dorsal as well as ventral structures. *No tail* (*ntl*) and *goosecoid* (*gsc*) are expressed in overlapping domains in the dorsal region of the shield stage embryos. As gastrulation progresses, the two domains separate with *gsc* moving anteriorly and *ntl* moving posteriorly [8]. *In situ* hybridization revealed that expression of *ntl* as well as *gsc* was reduced upon exposure to DTAB, thus indicating there is a reduction in both the anterior and posterior derived structures (Fig. 3G–J).

### DTAB activates Retinoic Acid signaling

The retinoic acid signaling pathway is known to play an important role in the anterior-posterior patterning of heart, brain, limbs, and somitic mesoderm [9] [10] [11] [12]. Since DTAB treated embryos exhibit defects in patterning consistent with a short A-P axis we assessed if RA-signaling is perturbed by DTAB.

Retinoic acid is synthesized in the body by the action of Raldh2 (Retinaldehyde dehydrogenase2). *Raldh2* is under the negative feedback regulation of RA. Further regulation of RA levels in the tissue is carried out by Cyp26s, RA metabolizing enzymes of the cytochrome P450 family that are induced by an increase in endogenous RA levels [13]. We analyzed the expression of *raldh2* by *in situ* hybridization and found that during gastrulation (80% epiboly) it is expressed in the dorsal side of the embryo in the region flanking the presumptive notochord (Fig. 3K–L). Treatment with DTAB caused a decrease in the *raldh2* transcript levels but the expression domains were maintained. Next we analyzed the expression of *cyp26a1* in zebrafish embryos. In the absence of any treatment, 6–8 somite stage embryos express high levels of *cyp26a1* in the tail bud. DTAB treated embryos exhibited an increase in the tail bud expression as well as in the anterior region of the developing brain (Fig. 3M–N). Taken together, these results suggest that there is an increase in RA signaling in DTAB treated embryos.

**Figure 4:**
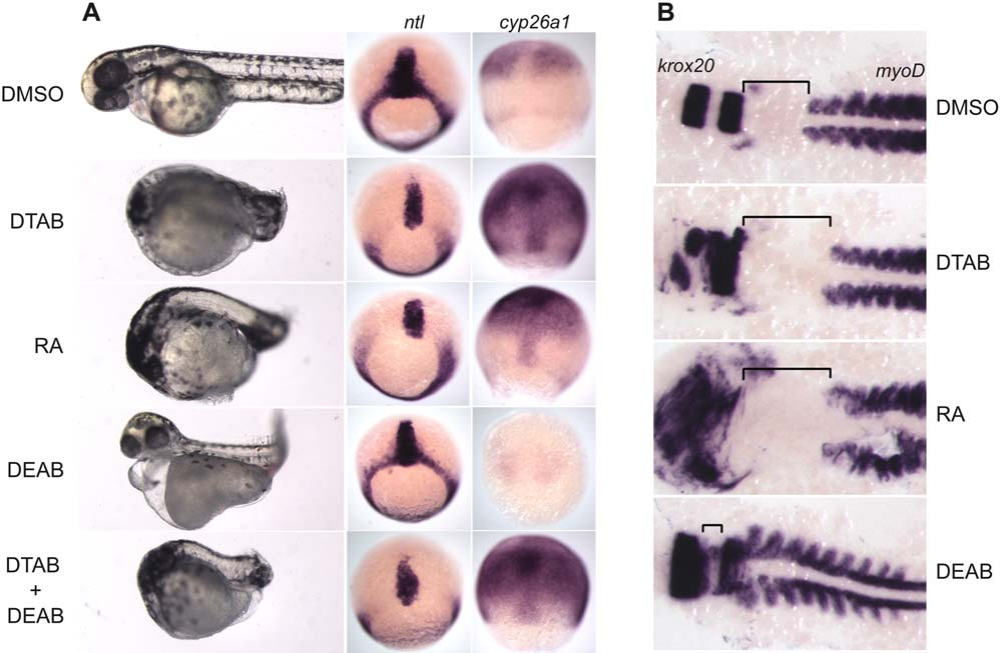
DTAB mimics effects of RA on early development. a) Treatment with DTAB, RA and DTAB+DEAB results in anterior-posterior shortened phenotype. DEAB alone caused pericardial edema, twisted body axis and downregulation of *cyp26a1*. DEAB treatment did not affect the *ntl* staining while RA and DTAB treatment led to a loss of *ntl* staining in the dorsal margin. DTAB, RA and DTAB+DEAB caused an increase in *cyp26a1* staining but DEAB alone caused downregulation of *cyp26a1*. Embryos were treated with DTAB (12.5 µM), RA (1 µM), DEAB (10 µM) and DTAB+DEAB or DMSO from 1hpf to 48hpf (for the bright field photography) and to 70% epiboly (for *in situ* hybridization for *ntl* and *cyp26a1*). Live bright field embryos are shown anterior to the left and dorsal to the top. *In situ* hybridized embryos, are shown, dorsal view with the animal pole to the top. b) DTAB and RA treatment caused an expansion in the distance between the *krox20* and *myoD* positive domains. DEAB caused a compression of the ‘neck’ region and led to a decrease in the distance between *krox20* and *myoD* domains. Embryos treated with DMSO, DTAB (1.6 µM), RA (0.125 µM) and DEAB (10 µM) were fixed at 9-somite stage and hybridized for *krox20* (rhombomeres 3 & 5) and *myoD* (somites). Following *in situ* hybridization the embryos were de-yolked and flattened under a coverslip for better visualization.

In order to understand the role of DTAB in RA signaling, we compared embryos treated with DTAB, *all trans*-RA, and DEAB, a small molecule inhibitor of Raldh2 activity. Embryos treated with DTAB or RA exhibited remarkably similar patterning defects leading to severely shortened anterior-posterior axis (Fig. 4A). DEAB treated embryos had a distinctive phenotype of smaller heads, curved body axis and pericardial edema. When *ntl* expression of DTAB and RA treated embryos were compared, they exhibited a similar loss of expression in the dorsal half of the margin. DEAB treated embryos showed no change in the *ntl* expression pattern. Expression of *cyp26a1* was significantly upregulated in the RA, as well as DTAB treated embryos when compared to vehicle treatment. DEAB treatment caused an almost complete loss of *cyp26a1* expression, which is consistent with its induction by RA signaling. In summary, DTAB and RA elicit similar responses in zebrafish embryos.

An increase in RA signaling, such as occurs in *giraffe* [14], a zebrafish mutant in the gene *cyp26a1*, causes an increase in the distance between the *krox20* (rhombomeres 3 and 5) and the *myoD* (somites) staining domains while the Raldh2 mutant *neckless* [15] shows a decrease in the distance between rhombomere 5 and the somites [14]. We compared the effects of RA (0.125 µM), DTAB (1.6 µM) and DEAB (10 µM) treatments on the distances between *krox20* and *myoD* domains in embryos. Consistent with previous reports, treatment with RA increased the distance between *krox20* and *myoD* markers when compared to vehicle treated embryos (Fig. 4B) while DEAB treatment reduced the distance significantly. DTAB treatment of embryos caused an increase in the distance between *krox20* and *myoD* staining, thus confirming that DTAB mimics RA in zebrafish embryos.

In order to understand if DTAB elicited its effects by stimulating endogenous RA synthesis, we treated embryos with both DTAB and DEAB. Co-treated embryos resembled embryos treated with DTAB alone (Fig. 4A). Expression of *ntl* and *cyp26a1* further confirmed that DTAB is able to induce RA signaling in embryos in the absence of Raldh2 function, thus indicating that DTAB activates RA signaling directly.

### DTAB activates RAR and RXR receptors

Retinoic acid is a lipophilic molecule that traverses the plasma membrane and binds to nuclear hormone receptors to activate target gene transcription [16]. There are two families of RA receptors known as RARs (Retinoic acid receptors) and RXRs (Rexinoid receptors) that transduce RA signals to activate transcription [17]. Differential expression and affinity of the RARs and RXRs for RA and its metabolites determine the different roles they play during development and disease [18] [19].

**Figure 5:**
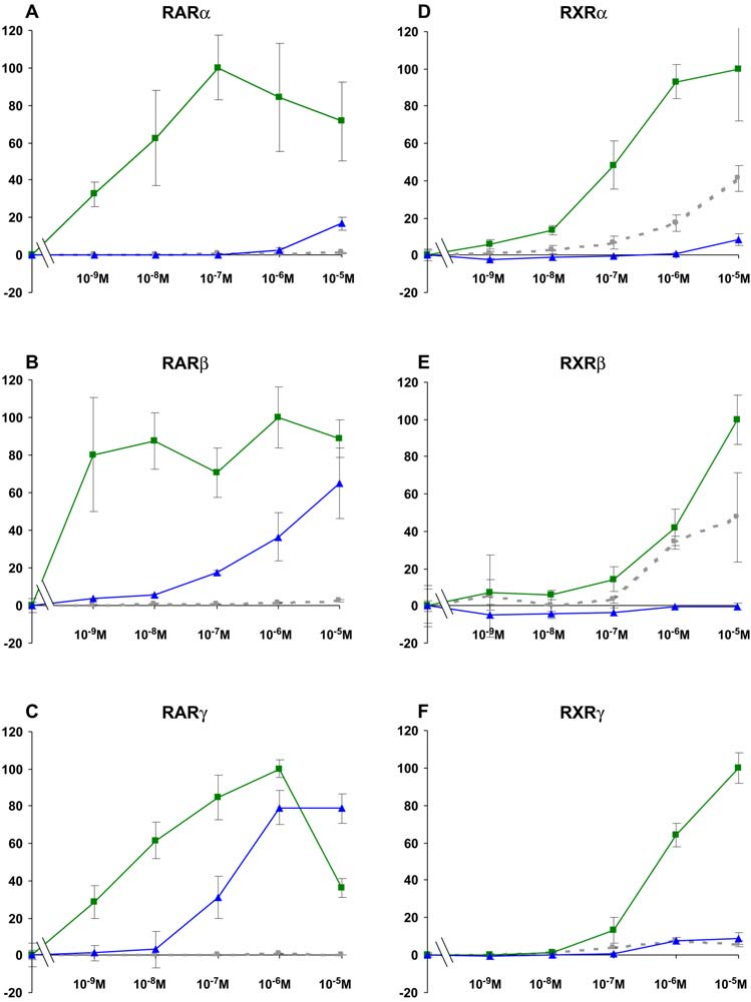
DTAB selectively activates RARβ and RARγ receptors. RA showed high efficiency in activating the RAR and RXR receptors. DTAB was a potent activator of RARβ and RARγ, but showed reduced efficiency towards RARα and RXRs. Hek293T cells were transfected with a luciferase reporter plasmid, a β-gal transfection-control plasmid and an RA receptor plasmid, as indicated. Cells were treated with 10^−9^ M to 10^−5^ M concentrations of DTAB or RA at 24 hours after transfection and harvested at 48 hours post transfection for luciferase and β-gal reporter assays. RA treated cells are shown by solid green squares and line and DTAB treated cells by solid blue triangles and line. Control cells transfected with only the luciferase and β-gal reporter plasmids (with no RA receptor plasmid) and treated with various concentrations of RA are shown by dashed gray line and gray circles. All luciferase readings were normalized to β-gal transfection controls. β-gal readings remained approximately equal throughout each experimental set showing equal transfection efficiency and cell viability. X-axis denotes RA or DTAB concentration. Y-axis represents arbitrary luminescence values. All RA and DTAB activities were normalized to the respective no-treatment controls. The maximum activity was set to 100% and all values calculated thereof.

Since DTAB mimics effects of RA on gene expression and embryo morphology we tested DTAB for RA receptor activation. We compared the ability of RA and DTAB to activate RAR and RXR receptors in a luciferase reporter based assay in Hek293T cells. As previously reported RA was found to be a potent activator of RARs and RXRs (Fig. 5). DTAB also activated a number of RARs and RXRs, but exhibited strongest activity toward RARγ, with significant activity toward RARβ. DTAB caused a very modest activation of RARα, RXRα, and RXRγ, and no significant activation of RXRβ at concentrations tested. Thus, we conclude that DTAB is a retinoid that exhibits differential selectivity for RARγ over all the other receptors tested.

## Discussion

Forward genetic screens in flies, worms, and zebrafish have provided useful insights into the processes of patterning and embryonic development. It has become increasingly evident that the same pathways that are involved in early development are also employed in controlling adult physiology, and that misregulation of these pathways leads to disease. Thus, small molecule modulators of genetic networks involved in early development can be instrumental in understanding and designing therapeutics for adult diseases. This connection between developmental defects and discovery of novel therapies is well demonstrated by the use of cyclopamine, a hedgehog signaling antagonist, in prostate [20], gastric [21], and breast cancer [22] therapies. Cyclopamine was serendipitously discovered as a plant compound that caused cyclopia in the progeny of sheep that grazed on the plants [23]. We have previously employed the same reasoning in identifying dorsomorphin, a novel small molecule inhibitor of BMP signaling, that caused dorsalization in zebrafish embryos and shows promise in developing therapeutics for anemia of chronic disease [24].

In order to uncover the richness of biological activities within existing chemical libraries, we performed a broad, phenotype-based screen on zebrafish embryos and identified numerous small molecules that perturbed a wide variety of different processes during development. We also observed that all the bioactive compounds identified in this screen clustered into a group with higher hydrophobicity when compared to the uniform distribution of hydrophilic and hydrophobic compounds in the whole library. We speculate that the increased hydrophobicity of compounds increases their permeability in zebrafish and that designing libraries enriched for compounds with log P values >0 could increase the ‘hit-rates’ of such libraries in zebrafish assays. However, it is worth noting that using zebrafish-based screens exclusively for small molecule discovery would preclude the identification of hydrophilic compounds with potentially interesting biological activities.

Our broad, high-content screen identified compounds affecting development of heart, blood, vasculature, brain, and body-axis. However, identifying target pathways of small molecules that cause complex phenotypes is often a challenge. To illustrate the strength of zebrafish in target identification, we selected DTAB, which causes anterior-posterior patterning defects in embryos, as a test case. As the retinoic acid pathway is known to be involved in A–P patterning, we deduced and were able to confirm that DTAB indeed functions as a retinoid. Thus, we demonstrate that the use of previously characterized mutant phenotypes and known roles of pathways in zebrafish embryogenesis can expedite the identification of molecular targets of small molecules.

Retinoic acid, a product of Vitamin A (retinol) metabolism, is a very important regulator of development and differentiation. In the vertebrate embryo, RA plays a crucial role in a variety of processes such as patterning of the anterior-posterior axis [25] and development of organs including heart [26], brain [10], and limb [11]. RA and its metabolites also play a crucial role in adult physiological functions such as promoting growth and differentiation, regulating apoptosis, and maintaining homeostasis in numerous tissues [27]. These pleiotropic roles of RA have been successfully harnessed for treatment of dermatological diseases as well as cancer therapy [27] [28]. RA is the first successful targeted drug for cancer and is still used to treat acute promyelocytic leukemia (APL) [27]. However, the success of RA treatment is limited by the rapidity with which RA is degraded in the body [29] and the pleiotropic effects of RA on the organism that lead to non-specific toxicity and retinoic acid syndrome [30]. Hence, discovery of retinoids with resistance to metabolism and/or restricted activity on different receptors, is an area of intense interest [29], [31], [32].

Interestingly, we found that DTAB exhibits selective activity towards RARγ and to a lesser extent towards RARβ, compared to the broad activity of RA. RARγ is predominantly expressed in the skin in the adult organism [19]. However, a number of other retinoid receptors are also expressed in the skin, the most abundant being RARτ. Psoriasis, a skin disease has been successfully treated with Tazarotene, a retinoid with selective activity towards RARβ and RARγ [32] [33], indicating a specific role for RARγ and/or RARβ in the disease. DTAB, with its selective activation of RARβ and RARγ could potentially be used to develop alternative therapies for this condition.

Decreased RARγ activity has been implicated in ovarian cancer, and AHPN (CD437), an RARγ agonist, has been shown to induce apoptosis and inhibit growth of ovarian tumor cell lines as well as transplanted tumors in nude mice [34], [35]. RARγ is also expressed in primitive hematopoietic stem cells (HSC). Recent studies show that mice null for RARγ have reduced numbers of HSCs and that activation of RARγ *ex vivo* can induce HSC self-renewal [36]. RARγ null mice also exhibit a non-HSC-autonomous myeloproliferative syndrome (MPS) [37]. Thus, RARγ selective agonists may be useful in understanding and designing therapeutics for multiple diseases characterized by reduced activity of RARγ.

## Materials and Methods

### Zebrafish maintenance

For all experiments TUAB zebrafish lines were used. Fish embryos were incubated in non-buffered E3.

### Small molecule screening

Zebrafish embryos at ∼1hpf were arrayed in round-bottomed 96-well plates in 100 µl of E3. Each 384-well plate of Chembridge diverse set E compounds (in DMSO) was transferred to four 96-well plates. The compounds from the 96-well plates were pin-transferred (approx. 100 nl) into wells with arrayed embryos. Embryos were incubated with the compounds in a humidified box at 28°C and were screened for phenotypes at 24hpf, 48hpf and 72hpf under a dissecting microscope. Compounds of interest were re-tested in 24-well plates.

### Partition coefficient analysis

Log P values of the whole tested library were calculated from values provided by Chembridge.

### Chemical compounds

All-*trans*-retinoic acid (RA) and 4-Diethylaminobenzaldehyde DEAB) were purchased from Sigma. Stock solutions of RA (1 mM) and DEAB (10 mM) were prepared in DMSO. DTAB (5133623) was purchased from Chembridge Corporation and a 12.5 mM stock was made in DMSO.

### Whole-mount *in situ* hybridization

Whole-mount *in situ* hybridization was performed essentially as previously described [38] using probes *krox20* and *myoD* [15], *cyp26a1* and *raldh2* [39], *goosecoid* (*gsc*) and *no tail* (*ntl*) [8] and *pax2a* [40]. For each experiment with different treatments of compounds, hybridization was carried out in parallel and color development was allowed to run for the same amount of time for all samples. Digoxigenin-labelled RNA antisense probes were generated with Promega RNA transcription reagents.

### Receptor activity assays

Hek293T cells were grown in DMEM containing 10% de-lipidated fetal calf serum for 24 hours and were transfected with either [pCMX-Gal-L-hRAR (with Gal4 DNA binding domain-RAR Ligand binding domain)+tk-px3-luc+tk-βgal] or [pCMX-mRXR+tk-apoA1-luc+tk-βgal]. ‘No-receptor’ controls were transfected with [tk-luc+tk-βgal] and treated with various concentrations of RA. 24 hours after transfection the cells were treated with RA, DTAB or left untreated. Cell lysates collected at 48 hours post-transfection were assayed for luciferase activity using the Promega luciferase reporter kit. The assay results were corrected against β-gal activity assayed using Promega β-gal reporter kit. All the reported results are from assays done in triplicates and repeated at least once. All the plasmids were kind gifts from Ronald M. Evans [41] or derived from those plasmids by cDNA subcloning.

## References

[1] Ortholand JY, Ganesan A (2004). Natural products and combinatorial chemistry: back to the future.. Curr Opin Chem Biol.

[2] Savchuk NP, Balakin KV, Tkachenko SE (2004). Exploring the chemogenomic knowledge space with annotated chemical libraries.. Curr Opin Chem Biol.

[3] Schreiber SL (2000). Target-oriented and diversity-oriented organic synthesis in drug discovery.. Science.

[4] Mayer TU, Kapoor TM, Haggarty SJ, King RW, Schreiber SL (1999). Small molecule inhibitor of mitotic spindle bipolarity identified in a phenotype-based screen.. Science.

[5] Kawasumi M, Nghiem P (2007). Chemical genetics: elucidating biological systems with small-molecule compounds.. J Invest Dermatol.

[6] MacRae CA, Peterson RT (2003). Zebrafish-based small molecule discovery.. Chem Biol.

[7] Mullins MC, Hammerschmidt M, Kane DA, Odenthal J, Brand M (1996). Genes establishing dorsoventral pattern formation in the zebrafish embryo: the ventral specifying genes.. Development.

[8] Schulte-Merker S, Hammerschmidt M, Beuchle D, Cho KW, De Robertis EM (1994). Expression of zebrafish goosecoid and no tail gene products in wild-type and mutant no tail embryos.. Development.

[9] Xavier-Neto J, Rosenthal N, Silva FA, Matos TG, Hochgreb T (2001). Retinoid signaling and cardiac anteroposterior segmentation.. Genesis.

[10] Begemann G, Meyer A (2001). Hindbrain patterning revisited: timing and effects of retinoic acid signalling.. Bioessays.

[11] Robert B, Lallemand Y (2006). Anteroposterior patterning in the limb and digit specification: contribution of mouse genetics.. Dev Dyn.

[12] Diez del Corral R, Olivera-Martinez I, Goriely A, Gale E, Maden M (2003). Opposing FGF and retinoid pathways control ventral neural pattern, neuronal differentiation, and segmentation during body axis extension.. Neuron.

[13] Ross AC (2003). Retinoid production and catabolism: role of diet in regulating retinol esterification and retinoic Acid oxidation.. J Nutr.

[14] Emoto Y, Wada H, Okamoto H, Kudo A, Imai Y (2005). Retinoic acid-metabolizing enzyme Cyp26a1 is essential for determining territories of hindbrain and spinal cord in zebrafish.. Dev Biol.

[15] Begemann G, Schilling TF, Rauch GJ, Geisler R, Ingham PW (2001). The zebrafish neckless mutation reveals a requirement for raldh2 in mesodermal signals that pattern the hindbrain.. Development.

[16] Clagett-Dame M, DeLuca HF (2002). The role of vitamin A in mammalian reproduction and embryonic development.. Annu Rev Nutr.

[17] Bastien J, Rochette-Egly C (2004). Nuclear retinoid receptors and the transcription of retinoid-target genes.. Gene.

[18] Mark M, Ghyselinck NB, Chambon P (2006). Function of retinoid nuclear receptors: lessons from genetic and pharmacological dissections of the retinoic acid signaling pathway during mouse embryogenesis.. Annu Rev Pharmacol Toxicol.

[19] Rees J (1992). The molecular biology of retinoic acid receptors: orphan from good family seeks home.. Br J Dermatol.

[20] Shaw A, Bushman W (2007). Hedgehog signaling in the prostate.. J Urol.

[21] Katoh Y, Katoh M (2005). Hedgehog signaling pathway and gastric cancer.. Cancer Biol Ther.

[22] Katano M (2005). Hedgehog signaling pathway as a therapeutic target in breast cancer.. Cancer Lett.

[23] Keeler RF, Binns W (1968). Teratogenic compounds of Veratrum californicum (Durand). V. Comparison of cyclopian effects of steroidal alkaloids from the plant and structurally related compounds from other sources.. Teratology.

[24] Yu PB, Hong CC, Sachidanandan C, Babitt JL, Deng DY (2008). The small molecule dorsomorphin inhibits BMP signals required for embryogenesis and iron metabolism.. Nat Chem Biol. In press.

[25] Schier AF, Talbot WS (2005). Molecular genetics of axis formation in zebrafish.. Annu Rev Genet.

[26] Wagner M, Siddiqui MA (2007). Signal transduction in early heart development (II): ventricular chamber specification, trabeculation, and heart valve formation.. Exp Biol Med (Maywood).

[27] Altucci L, Gronemeyer H (2001). The promise of retinoids to fight against cancer.. Nat Rev Cancer.

[28] Stadler R, Kremer A (2006). Therapeutic advances in cutaneous T-cell lymphoma (CTCL): from retinoids to rexinoids.. Semin Oncol.

[29] Njar VC, Gediya L, Purushottamachar P, Chopra P, Vasaitis TS (2006). Retinoic acid metabolism blocking agents (RAMBAs) for treatment of cancer and dermatological diseases.. Bioorg Med Chem.

[30] Asou N (2007). 2. All-trans retinoic acid in the treatment of acute promyelocytic leukemia.. Intern Med.

[31] Altucci L, Leibowitz MD, Ogilvie KM, de Lera AR, Gronemeyer H (2007). RAR and RXR modulation in cancer and metabolic disease.. Nat Rev Drug Discov.

[32] Weindl G, Roeder A, Schafer-Korting M, Schaller M, Korting HC (2006). Receptor-selective retinoids for psoriasis: focus on tazarotene.. Am J Clin Dermatol.

[33] Chandraratna RA (1997). Tazarotene: the first receptor-selective topical retinoid for the treatment of psoriasis.. J Am Acad Dermatol.

[34] Holmes WF, Dawson MI, Soprano RD, Soprano KJ (2000). Induction of apoptosis in ovarian carcinoma cells by AHPN/CD437 is mediated by retinoic acid receptors.. J Cell Physiol.

[35] Langdon SP, Rabiasz GJ, Ritchie AA, Reichert U, Buchan P (1998). Growth-inhibitory effects of the synthetic retinoid CD437 against ovarian carcinoma models in vitro and in vivo.. Cancer Chemother Pharmacol.

[36] Purton LE, Dworkin S, Olsen GH, Walkley CR, Fabb SA (2006). RARgamma is critical for maintaining a balance between hematopoietic stem cell self-renewal and differentiation.. J Exp Med.

[37] Walkley CR, Olsen GH, Dworkin S, Fabb SA, Swann J (2007). A microenvironment-induced myeloproliferative syndrome caused by retinoic acid receptor gamma deficiency.. Cell.

[38] Aerne B, Ish-Horowicz D (2004). Receptor tyrosine phosphatase psi is required for Delta/Notch signalling and cyclic gene expression in the presomitic mesoderm.. Development.

[39] Dobbs-McAuliffe B, Zhao Q, Linney E (2004). Feedback mechanisms regulate retinoic acid production and degradation in the zebrafish embryo.. Mech Dev.

[40] Thisse B, Pflumio S, Fürthauer M, Loppin B, Heyer V, Degrave A, Woehl R, Lux A, Steffan T, Charbonnier XQ, Thisse C (2001). Expression of the zebrafish genome during embryogenesis (NIH R01 RR15402).. ZFIN Direct Data Submission.

[41] Mangelsdorf DJ, Borgmeyer U, Heyman RA, Zhou JY, Ong ES (1992). Characterization of three RXR genes that mediate the action of 9-cis retinoic acid.. Genes Dev.

